# A randomized controlled cross-over trial investigating the effect of anti-inflammatory diet on disease activity and quality of life in rheumatoid arthritis: the Anti-inflammatory Diet In Rheumatoid Arthritis (ADIRA) study protocol

**DOI:** 10.1186/s12937-018-0354-x

**Published:** 2018-04-20

**Authors:** Anna Winkvist, Linnea Bärebring, Inger Gjertsson, Lars Ellegård, Helen M. Lindqvist

**Affiliations:** 1 Department of Internal Medicine and Clinical Nutrition, Institute of Medicine, Sahlgrenska Academy, University of Gothenburg, Box 459, S-405 30 Gothenburg, Sweden; 2 Department of Rheumatology and Inflammation Research, Institute of Medicine, Sahlgrenska Academy, University of Gothenburg, Gothenburg, Sweden; 3 Clinical Nutrition Unit, Department of gastroenterology, Sahlgrenska University Hospital, Gothenburg, Sweden

**Keywords:** Rheumatoid arthritis, Diet, Anti-inflammatory, Quality of life, Sweden

## Abstract

**Background:**

Rheumatoid arthritis (RA) is a chronic inflammatory disease that affects 0.5–1.0% of the population, and where many patients in spite of modern pharmacological treatment fail to reach remission. This affects physical as well as mental wellbeing and leads to severely reduced quality of life and reduced work capacity, thus yielding high individual as well as societal costs. As a complement to modern pharmacological treatment, lifestyle intervention should be evaluated as a treatment option. Scientific evidence exists for anti-inflammatory effects by single foods on RA, but no study exists where these foods have been combined to obtain maximum effect and thus offer a substantial improvement in patient life quality. The main goal of the randomized cross-over trial ADIRA (Anti-inflammatory Diet In Rheumatoid Arthritis) is to test the hypothesis that an anti-inflammatory diet intervention, compared to a regular diet, will decrease disease activity and improve quality of life in patients with stable established RA.

**Methods:**

In total, 50 RA patients with moderate disease activity are randomized to receive initially either a portfolio diet based on several food items with suggested anti-inflammatory effects or a control diet during 2 × 10 weeks with 3 months wash-out between diets. Food bags are delivered weekly by a home food delivery chain and referred to as the fiber bag and the protein bag, respectively, to partially blind participants. Both groups continue with regular pharmacological treatment. Known food biomarkers will be analyzed to measure intervention compliance. Impact on disease severity (measured by DAS28, a composite score which predicts disability and progression of RA), risk markers for cardiovascular disease and quality of life are evaluated after each diet regimen. Metabolomics will be used to evaluate the potential to predict responders to dietary treatment. A health economic evaluation is also included.

**Discussion:**

The nutritional status of patients with RA often is poor and many ask their physician for diet advice. No evidence-based dietary guidelines for patients with RA exist because of the paucity of well-conducted sufficiently large diet intervention trials. ADIRA is an efficacy study and will provide evidence as to whether dietary treatment of RA can reduce disease activity and improve quality of life as well as reduce individual and societal costs.

**Trial registration:**

ClinicalTrials.gov Registration Number: NCT02941055.

**Electronic supplementary material:**

The online version of this article (10.1186/s12937-018-0354-x) contains supplementary material, which is available to authorized users.

## Background

Rheumatoid arthritis (RA) is a chronic autoimmune disease, characterized by systemic inflammation and joint damage. In spite of modern expensive pharmacological treatment, much pain and suffering as well as reduced work capacity remain. Hence, additional treatment options such as diet interventions are requested by patients and treating physicians.

RA affects 0.5–1% of the population globally [1]. The inflammation leads to joint destruction and fatigue as well as to impaired physical functioning, work productivity and activities of daily living, and compromise overall emotional well-being. A negative impact on relationships with friends and family has been reported by one-fifth of RA patients. Further, suboptimal mental health is often reported [2]. The inflammation also leads to increased risk for cardiovascular diseases (CVD) and together with the immune-suppressive treatment also to increased risk for infections--the two leading causes of death among patients with RA [3]. Patients with RA face approximately 50% increased risk of myocardial infarction and stroke compared to the general population and their risk of CVD is comparable to that of patients with diabetes [4, 5]. Consequently, life expectancy among patients with RA is reduced by 5–10 years, compared to non-diseased individuals.

Modern RA therapy includes immunosuppression by disease modifying anti rheumatic drugs (DMARD), steroids and the more recent line of biological therapies such as TNF-α-inhibitors. The treatment aims at long-term remission i.e. no pain, tenderness of joints or functional impairment. This is achieved in 10–50% of patients with early RA [6]. However, a recent systematic review highlighted that, in spite of these modern therapies, a substantial proportion of patients with RA still experience severely impaired health [2].

Thus, in spite of modern therapy, RA continues to present a considerable human and economic burden. An estimated one-third of patients with RA terminate employment prematurely, and 5 years after diagnosis 30–40% of patients experience work disability [2]. The authors of the systematic review conclude that “*Despite advances in treatment that have helped to improve outcomes for patients with RA, treatment goals, aspirations, and expectations are seldom met for both patients and physicians. Novel treatment approaches for RA need to be tested for their ability to ameliorate contemporary unmet need.*” [2]

Associations between diet and chronic diseases such as CVD, cancer and diabetes have long been established [7, 8]. Hence, for these diseases evidence-based dietary treatment guidelines are available. In contrast, for inflammatory diseases such as RA no dietary guidelines exist, reflecting the ambiguous evidence-base. Currently, patients with RA in Sweden are encouraged to follow the same recommendations as the general population. These aim at increasing intake of fruit and vegetables to 500 g/day, fish and shellfish to 2–3 times per week, choosing whole grain over refined grain products and low-fat dairy products over full-fat varieties. Also, intake of red meat and refined sugars should be reduced [9].

The potential for diet to improve RA is obvious; diet could influence symptoms of RA by influencing the inflammatory activity, changing the lipid profile, increasing antioxidant levels, and altering the microflora of the intestine. Observational studies show lower incidences of RA among people with a healthy diet as captured by the Healthy Eating Index [10], larger consumption of LC n-3 PUFA [11] and fish [12]. Intervention studies using complete diets show that the Mediterranean diet (characterized by high intakes of olive oil and fish and low intake of red meat and dairy products), low-fat vegan diet and diets rich in unsaturated fat or probiotics have positive effects at alleviating pain and on inflammation markers [13]. Fish oil or dietary LC n-3 PUFA, i.e. reflecting fish intake, in addition to treatment with DMARD have been reported to increase the number of successful and continued DMARD treatments, indicating that pharmacological and dietary treatment complement each other [14, 15]. So far, no study has combined all components with indicative effects on RA, thus limiting the possibility to evaluate the full potential of dietary treatment of the disease. In sum, high-quality studies are needed that evaluate the combined effect of foods rich in anti-inflammatory and immune strengthening substances that could interact synergistically on RA.

Whether patients with RA respond to a dietary intervention or not might relate to genetics, habitual diet, the microbiome and/or the diversity and intensity of disease activity. Metabolomics, ie the analysis of low molecular weight metabolites, investigates overall metabolomic activity, taking genetic and environmental variation into account. It is a powerful tool to measure global and dynamic metabolic responses in disease and clinical intervention and some research also exist on RA. Different baseline metabolic profiles have been identified among patients with active RA compared to those in remission [16]. Thus, it may be possible to identify metabolic biomarkers to predict response also to dietary treatment among patients with RA. The metabolomics methods are relatively new and therefore few dietary interventions have used this approach to look at metabolic effects. Further, since the metabolism might be pertubated in patients with RA due to the metabolic syndrome or cachexia, metabolomics opens new possibilities to look at previously unknown metabolic changes as a consequence of a specific diet.

### Research objective and specific questions

We hypothesize that an anti-inflammatory diet, in addition to pharmacological treatment, can improve health and quality of life in patients with rheumatoid arthritis. Specific questions are if an anti-inflammatory diet, compared to a usual Swedish diet, can reduce inflammation, pain and disease activity; reduce risk markers of cardiovascular disease; improve body composition; improve quality of life; reduce patient and societal costs related to the disease, and if metabolomics can be used to predict responders.

## Methods/Design

The randomized controlled cross-over trial ADIRA (Anti-inflammatory Diet In Rheumatoid Arthritis) is carried out among patients with established RA with moderate disease activity to evaluate response to a portfolio diet treatment, compared to control diet (i.e. western diet). Both groups continue with pharmacological treatment as usual but herbal medicines or diet supplements are not allowed. In Sweden, patients with established RA are treated with DMARD and biologics at out-patient clinics according to the Swedish Society for Rheumatology Guidelines [17]. The portfolio diet treatment builds on a combination of individual food items with indicative effects on different RA symptoms. The trial thus evaluates the treatment potential of a diet based on a combination of functional concepts that likely potentiate each other in their anti-inflammatory effects. Although it is not possible to fully blind a diet intervention, the two groups are treated as similarly as possible except for diet content. Assessors of outcome are blinded. With respect to blinding of participants, see below.

**Fig. 1:**
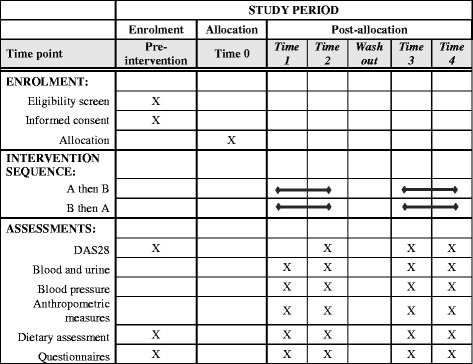
Content for the schedule of enrolment, intervention and measurements according to SPIRIT requirements

**Fig. 2:**
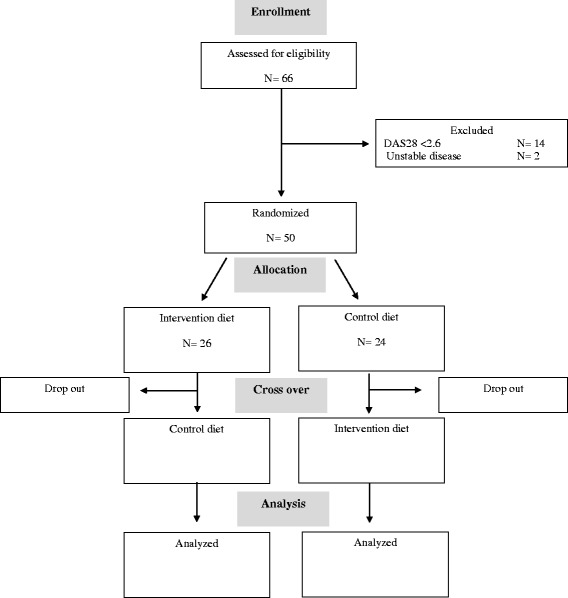
Flow diagram of participant recruitment during the cross-over trial according to CONSORT

The schedule of enrolment, intervention and measurements according to Standard Protocol Items: Recommendations for Intervention Trials (SPIRIT) requirements is shown in Fig. 1, and the SPIRIT Checklist is available as Additional file 1. The cross-over study is presented in Fig. 2.

### Participant selection

Invitation letters to the study were sent to patients with RA residing in the Västra Götaland region, Sweden, identified through the Swedish Rheumatology Quality Register, SRQ (http://srq.nu/). Responders to the invitation letters or to posters at the Sahlgrenska University Hospital were invited to screening visits. Inclusion criteria was disease duration > 2 years, active disease i.e. DAS28 ≥ 2.6 that was clinically stable and under adequate control and medication at the screening visit. DAS28 stands for Disease Activity Score, where swelling and tenderness in 28 different joints are evaluated in combination with information on erythrocyte sedimentation rate (ESR) as well as patient-reported global assessment of health [18]. Exclusion criteria included other condition demanding active medical attention, changes in DMARD during last 8 weeks, intolerant or allergic to food items included in the intervention diets or not willing to eat omnivore diet. Between February and September 2017, 13 men and 53 women, 18–70 years of age, were screened for eligibility. At the screening visit, study details were presented, informed consent obtained and habitual diet was assessed. All ADIRA study visits take place at the research ward of the Clinical Rheumatology Research Centre, Sahlgrenska University Hospital, Gothenburg, Sweden, and measurements are carried out by two registered nurses experienced in rheumatology assessments along with the project team.

Patients agreeing to participate and fulfilling all inclusion criteria were thereafter invited to baseline measurements within a few weeks. These included clinical phenotype, body composition (bioelectric impedance spectroscopy) and health assessment, 3 day food record, serum samples for blood lipids, urine sample, metabolomics and inflammation markers (e.g., high-sensitive C-reactive protein (hs-CRP), cytokines and intercellular adhesion molecules), Health Assessment Questionnaire [19] (HAQ), Quality of Life (QoL) and a questionnaire on socio-demographics, lifestyle, acute health care visits and non-steroidal anti-inflammatory drugs (NSAID) and corticosteroid consumption.

Thereafter, patients were randomized to either diet regimen for 10 weeks. Randomization scheme was computer-generated and revealed to participants and study team at the baseline visit. At the end of the 10 wk. period, the same measurements are repeated. Individual pharmacological treatment is reported at baseline and any changes or additions in pharmacological treatment are noted during the study period. After a 2–3-mo wash-out period, the alternative diet regimen is followed for another 10 weeks. Compliance to the diets is monitored by a telephone administered interview at wk. 5 in both diet periods and with analyses of plasma phospholipid LC n-3 PUFA at the end of each diet period as well as with 3 d food record at the end of each diet period. Support for participants is provided at this telephone interview to promote trial retention; it is also possible for participants to contact the study team at any time during the intervention. Participants are asked to report any side effects. Should a participant decide to quit the trial, no further data are collected.

### Diet intervention

For both diets, food bags are delivered weekly by a home food delivery chain and make up approximately 50% of the daily intake during 5 weekdays. All meals are easily prepared with minimal peeling and cutting that could be challenging during active phases of RA. The meals are varied in style and ingredients to promote compliance and to ensure satisfactory nutrient intake. Participants in both groups are provided with recipes and information on the diet they are to follow. All participants are encouraged to maintain weight stability. Macronutrient composition of both diets is presented in Table 1.

**Table 1:** Macronutrient content of the two intervention diets in ADIRA

	Fiber diet		Protein diet	
Macronutrient	Content	% of total energy	Content	% of total energy
Energy (kJ)	4623		4596	
Energy (kcal)	1105		1098	
Carbohydrate (g)	119	48	129	49
Protein (g)	46	17	62	23
Fat (g)	44	35	34	28
SFA (g)	12	9	16	13
MUFA (g)	14	11	11	9
PUFA (g)	14	11	11	3
Omega 3 (g)	4	3	0.8	0.6

*SFA* saturated fatty acids, *MUFA* mono saturated fatty acids, *PUFA* poly-unsaturated fatty acids

The anti-inflammatory diet bag contains 5 main meals, 5 breakfasts and 5 in-between-meal snacks per week: main meal dishes includes fish 3–4 times weekly, mainly oily fish, and 1–2 vegetarian meals rich in prebiotics (dietary fiber). Breakfast meals consist of low-fat dairy products, whole grains, nuts, berries and probiotic fruit juice is included with each breakfast meal. In-between-meal snacks comprise of 2 fruits per day. Participants are also provided with olive/rapeseed oil for cooking. Wholegrain, legumes and vegetables ensure an adequate intake of fiber and prebiotics, and fish, oils and nuts provide unsaturated and LC n-3 PUFA and probiotics are added to the diet. Participants are not provided with any meat and are encouraged to restrict intake of red meat to 3 servings per week. This diet therefore combines components of a vegetarian and Mediterranean diet with LC n-3 PUFA and probiotics, which all have shown promising effects on clinical outcomes of RA. Total content from animal sources (fish and low fat dairy products) is 28% and from vegetable sources 72%. In an effort to blind the intervention to participants, this bag is referred to as *the fiber bag* instead of anti-inflammatory bag.

The control diet bag contains 5 main meals, 5 breakfasts and 5 in-between-meal snacks per week: five dishes including meat or chicken. The 5 servings of breakfast meals consist of full-fat dairy products, corn flakes, white bread and fruit juice without probiotics. In-between-meal snacks comprise of curd and protein bars. Participants will also be provided with a mix of butter and margarine for spread, together with recipes and instructions for food preparation. To blind the intervention to participants, this bag is referred to as *the protein bag*. Protein content is not unusually high; it is actually similar to the reported intakes of Swedish men and women between the ages of 45–65 years in the national dietary survey Riksmaten 2010 in terms of macronutrients and quality of fat and carbohydrate intake [20]. Thus, the diet is representative of the dietary habits of the average Swedish person. Total content from animal sources (beef and chicken) is 48% and from vegetable sources 52%. In Sweden, high protein and full fat products rich in saturated fat (e.g, butter) have been trendy during the last years and it is not obvious to most participants which diet is the healthy one. Also, they are informed that the effects of two different diets are evaluated in the study.

Participants expressing dislike of a certain food item have the possibility to exchange this for a food item of similar nutrient composition.

### Analysis of outcome

Outcomes are measured after each diet regimen. Primary outcome is DAS28, where we expect a difference between diet regimens > 0.6 units (considered clinically relevant) in the intention-to-treat analysis. Secondary outcomes include DAS28-CRP (where hs-CRP is used instead of ESR in the DAS28 calculation), inflammation markers, anthropometry, QoL, HAQ, blood lipids, blood pressure, acute health care visits and NSAID and corticosteroid consumption. Differences in outcomes will be compared for the two diet regimens in ANCOVA analyses, adjusting for baseline disease activity scores and potential confounders (e.g., age, other lifestyle factors). Mixed models also will be applied to fully handle missing data. Interaction by sex and socio-economic position will be evaluated.

Drop-outs will be included in secondary analyses of changes in DAS28 within one diet period if they have completed at least one diet period. Drop-out analyses will be carried out in that baseline information will be compared between drop-outs and those who complete the study.

Data will be entered and stored at the Dept Internal Medicine and Clinical Nutrition, Sahlgrenska Academy, University of Gothenburg, Sweden. Stored data files will not contain any personal identifying information; only the PI will have the code list and this will be kept in a locked drawer. Quality control procedures of data will be carried out by the PI. All members of the research team have access to final trial dataset. Results will be published in international peer-reviewed journals. They will also be shared with professional societies in rheumatology and nutrition. Participants will receive information about their own results at the end of the trial.

### Cost-effectiveness

ADIRA is an efficacy study and hence not optimal for evaluation of cost-effectiveness in clinical use. Like in all lifestyle interventions, the long lasting compliance to lifestyle changes is essential for such evaluations and this will be evaluated in the following effectiveness ADIRA trial. However, it will still be possible to evaluate if the ADIRA efficacy trial has *potential* to be cost-effective in clinical praxis. The main cost is incremental costs of anti-inflammatory diet compared to control diet and these exist as long as the new diet is consumed, but also participants’ time spent will be recorded and valued. Expected benefits include gain in QoL and reduced use of acute health care and medication as well as sick leave. Gain in QoL (mainly pain relief, measured with SF-36/SF-6D [21–23], EQ-5D and EQ VAS [24]) likely occurs in short time and is thus possible to evaluate 10 weeks from start. In contrast, changes in health care use and sick leave may take longer to accrue. Therefore, the main economic analysis will be a comparison between incremental food costs and incremental QoL while the dietary change is in use. In practice, the food costs and QoL gain per week diet is used, presented as costs per quality-adjusted life-years (QALY). Best evidence of the effect of pain relief for RA patients on use of acute health care, medication and sick leave will be obtained from literature and used in evaluations of the cost-effectiveness of anti-inflammatory diet compared to control diet.

### Sample size

To detect a DAS28 difference of 0.6 units (clinically relevant) with 90% power and α = 0.05, 38 patients are needed in cross-over design. Skoldstam [13] detected a difference of 0.6 units with 30 patients per group in a diet parallel trial. We have carried out a pilot cross-over diet study that detected a difference of 0.34 units with 23 patients, confirming that cross-over design reduces the sample size (unpublished results). To account for drop-out or non-compliance, ADIRA cross-over study included 50 women and men.

### Metabolomics

For metabolomics, serum samples will be analyzed with nuclear magnetic resonance (NMR) at the Swedish NMR Centre at University of Gothenburg, Sweden, as well as with mass spectrometry (MS) at Chalmers Technical Institute, Gothenburg, Sweden. We will use an untargeted metabolomics approach for a global description of the metabolites that can be found in participants’ serum using unsupervised statistical analyses, ie principal component analysis (PCA). Subsequently supervised clustering, ie orthogonal projection to latent structures-discriminant analyses (OPLS-DA [25]) and OPLS-EP [26] (for paired data) will be performed to compare metabolites between patients who respond and who don’t respond to the inflammatory diet. Finally, in a targeted metabolomics approach, metabolites providing the greatest discrimination between groups of responders vs. non-responders will be identified using publicly and commercially available as well as in-house developed databases.

### Ethical considerations

Ethical approval has been received from the Gothenburg Regional Ethical Review Board, Gothenburg, Sweden (agreement No: 976–16 of November 21, 2016).

## Discussion

The nutritional status of patients with RA often is poor, many are overweight or obese and many ask their physician for diet advice. However, no evidence based dietary guidelines for patients with RA exist because of the paucity of well-conducted sufficiently large diet intervention trials. Most existing studies are observational or focusing on few dietary items. ADIRA will contribute high-quality scientific evidence, as it is based on state-of-the art methodology in terms of diet composition and evaluation of compliance using biomarkers. It is original and innovative in that it applies a portfolio diet and home-delivery services. The portfolio diet treatment builds on a combination of individual food items with suggested effects on different RA symptoms that likely potentiate each other in their anti-inflammatory effects. The treatment likely has high cost benefit due to its low adverse event rate and low cost, in comparison with new and potent medications. It is an efficacy trial; hence internal validity is most crucial and great care is taken to achieve high internal validity. External validity, of secondary importance in this efficacy trial, can be evaluated because patients are recruited from the Swedish Rheumatology Quality Register. Our previous pilot trial involving patients with RA indicated that high external validity was possible to achieve.

### Strengths

ADIRA trial has several strengths. It has a cross-over design that minimizes individual variation between diet groups. The trial is population-based in that all eligible patients with RA in the region are identified through the Swedish Rheumatology Quality Register and invited to participate. Patients are randomized to a specific diet at the beginning of the study, meaning that effects of season, holidays and carry-over from previous test period should be evenly spread between the two diets. The intervention diets are developed by registered dieticians and have a similar content of macronutrients and energy. The food bags are delivered to participants’ homes by a home delivery food chain every week, which is convenient for participants. Also the meals are easy to prepare or ready meals, with a high nutritional quality. The food bags are referred to as the fiber bag and the protein bag to partially blind participants, and assessors of outcome are fully blinded.

The primary outcome measure, DAS28, is the most clinically relevant outcome and often used in double-blinded pharmacological interventions. The trial is carried out in collaboration with rheumatologists and, hence, results can easily be shared with treating physicians. Compliance to the diet is measured with objective food biomarkers. In addition, metabolomics will be used to identify biomarkers of response to the intervention, and an evaluation of the cost effectiveness of the trial also will be performed.

### Limitations

The trial also has some limitations. The disease is more common among women than men and this is mirrored in our study sample where men make up a minority. Further, the recruitment area is limited to Västra Götaland Region so that patients can visit the research clinic at Sahlgrenska University Hospital in Gothenburg and receive the home delivered food bags. To obtain the planned sample size within this area, broad inclusion criteria are applied. Hence, age varies between 27 and 74 years, disease duration is minimally 2 years and type of pharmacological drugs used by participants varies tremendously between patients and over time. Potential participants were invited by postal mail to patients in SRQ residing in Västra Götaland Region and posters at the Sahlgrenska University Hospital. Among the 66 patients booked for screening, 75% qualified to start the trial. Hence, ADIRA participants represent a selected group of patients with RA and potential selection bias has to be evaluated. Socio-demographic information on ADIRA participants will later be compared with similar information on patients in Sweden with rheumatoid arthritis as described in the Swedish National Patient Register [27]. The main outcome DAS28 includes both objective components such as erythrocyte sedimentation rate and number of swollen and tender joints, and subjective patient-relevant components such as the patient-reported global assessment of health. It is the most clinically relevant outcome but, it is partially subjective, which must be considered when interpreting results.

Also, participants only receive about 50% of their total dietary intake during 5 days of the week during each 10-wk diet period. They are instructed to add foods in line with the intervention diet, but we cannot fully know to what extent this is happening. Objective biomarkers of compliance are used together with a telephone interview half way through each diet period and a 3-d food record at the end of each diet period. This will only partially capture compliance. However, because ADIRA is a cross-over study each participant acts as his/her control. Likely, the home diet will be similar for each individual in both diet periods except for what is guided by the intervention and this will minimize the effect of the home diet on the intervention. In addition, it is possible that the 10 week long period of each diet may be too short to provide a measurable effect on disease severity. Finally, RA is a disease where activity varies over time and pharmacological treatment changes periodically. This will likely dilute any true effects on disease activity of one diet over the other. Still, evaluations of additional treatment for patients with RA are needed and should be performed while accounting for these methodological challenges.

## Conclusion

ADIRA is an efficacy study and will provide evidence whether dietary treatment of rheumatoid arthritis can reduce disease activity and improve quality of life as well as reduce individual and societal costs in addition to pharmacological treatment.

## Additional file

Additional file 1: SPIRIT 2013 Checklist: Recommended items to address in a clinical trial protocol and related documents*. (DOC 121 kb)

## Abbreviations

ADIRA: Anti-inflammatory Diet In Rheumatoid Arthritis;
BMI: body mass index
CVD: cardiovascular diseases
DAS28: Disease Activity Score
DMARD: disease modifying anti rheumatic drugs
HAQ: Health Assessment Questionnaire
MS: mass spectrometry
NMR: nuclear magnetic resonance
NSAID: non-steroidal anti-inflammatory drugs
OPLS-DA: orthogonal projection to latent structures-discriminant analyses
PCA: principal component analysis
PUFA: poly unsaturated fatty acids
QALY: quality-adjusted life-years
QoL: Quality of Life
RA: Rheumatoid arthritis
SPIRIT: Standard Protocol Items: Recommendations for Intervention Trials
SRQ: Swedish Rheumatology Quality Register
